# Acute Eosinophilic Pneumonia Associated With the Anti-COVID-19 Vaccine AZD1222

**DOI:** 10.7759/cureus.18959

**Published:** 2021-10-21

**Authors:** Amal Miqdadi, Mohammed Herrag

**Affiliations:** 1 Respiratory Medicine, Cheikh Khalifa Bin Zayed Al Nahyan Hospital, Casablanca, MAR; 2 Medicine, Mohammed 6 University of Health and Sciences, Casablanca, MAR

**Keywords:** eosinophilic pneumonia, acute respiratory failure, astrazeneca, azd1222, vaccine

## Abstract

SARS-CoV-2 is an emerging virus causing the contemporary global pandemic. No cure has yet been discovered. Therefore, vaccination remains the only hope. We report the case of a 66-year-old male patient with a history of allergies. Five hours after his vaccination with the anti-COVID-19 vaccine AZD1222 (ChAdOx1 nCoV-19, AstraZeneca), he developed acute respiratory distress. The biological assessment showed hyperleukocytosis, 20% of which are eosinophils. Diagnosis of severe postvaccination acute eosinophilic pneumonia was retained given the history of allergy, lack of improvement on antibiotics, elimination of all other probable causes of eosinophilia, and improvement on corticosteroids. Such reactions of eosinophilic pneumonia have only been described twice: once following vaccination with the influenza vaccine (Vaxigrip*) and the other after vaccination with the 23-valent pneumococcal polysaccharide vaccine (Pneumovax 23*). Hypereosinophilia must be taken into consideration, feared, and prevented. Although rare and severe, post-COVID-19 vaccination acute eosinophilic pneumonia remains well manageable with corticosteroids with a good outcome. Therefore, in some poorly monitored patients with allergy or asthma, the use of another less allergenic vaccine could be considered to avoid such reactions.

## Introduction

SARS-CoV-2 is an emerging virus and is the cause of the contemporary global pandemic. There remains no curative treatment for this biological agent that could cause a secondary acute respiratory distress syndrome. Therefore, vaccines are our only hope to end the pandemic. However, similar to any vaccination, certain complications have been reported, including thromboembolic complications [1,2], neurological complications [3], or even immuno-allergic reactions [4,5]. Thus, we present the first case report of a severe acute eosinophilic pneumonia following vaccination with the Covishield* (AZD1222, ChAdOx1 nCoV-19, AstraZeneca) COVID-19 vaccine.

## Case presentation

**Figure 1:**
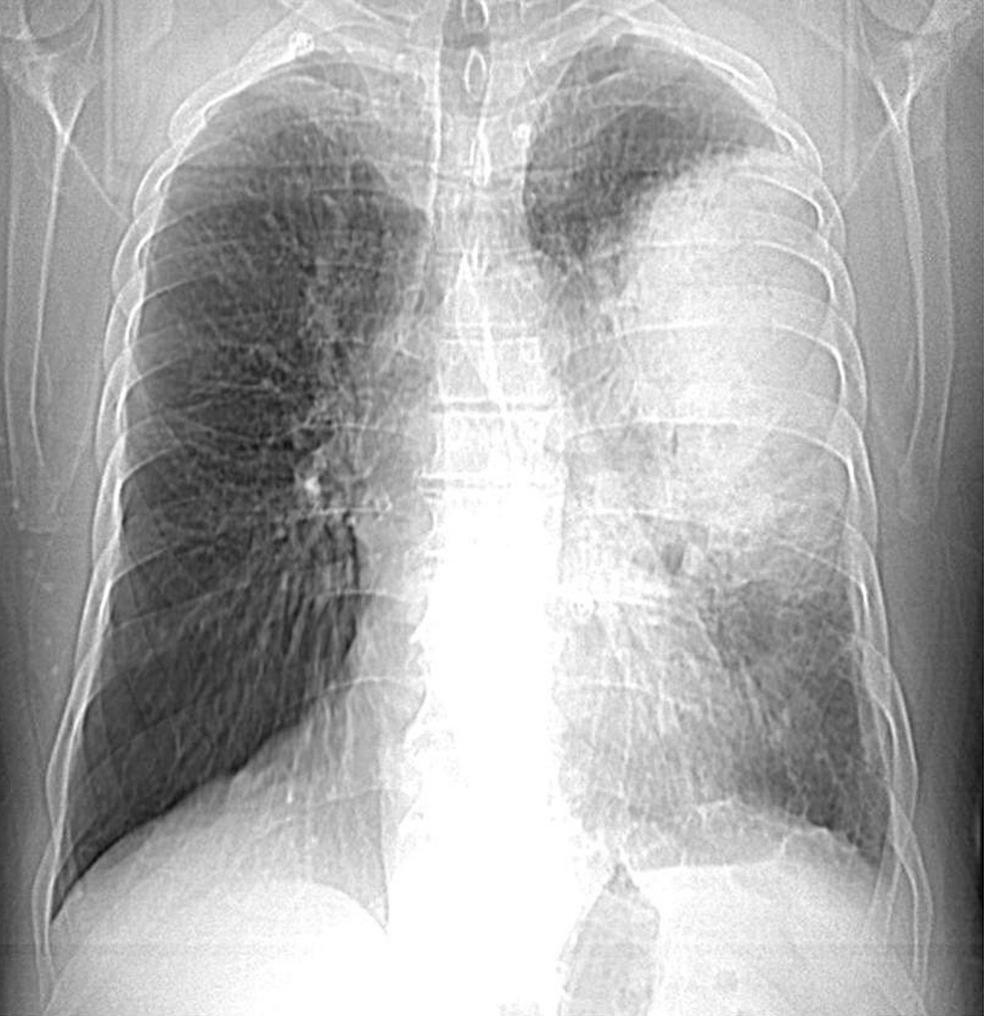
First chest X-ray showing alveolitis type shadowing taking up two-thirds of the left lung

Our patient was a 66-year-old professional painter with a history of poorly monitored allergic rhinoconjunctivitis following exposure to strong odors and dust mites without any notion of drug or food allergies nor a recent drug introduction. He had never traveled to the tropics and neither been treated for parasitosis. Five hours after his first dose of the anti-COVID-19 vaccine AZD1222, he presented with chest tightness with wheezing, polypnea, fever, asthenia, and muscle weakness. A chest X-ray showed alveolitis type shadowing taking up two-thirds of the left lung during a general visit (Figure 1). No clinical improvement had occurred although he was treated with amoxicillin/clavulanic acid by a general practitioner for supposed bacterial pneumonia.

**Figure 2:**
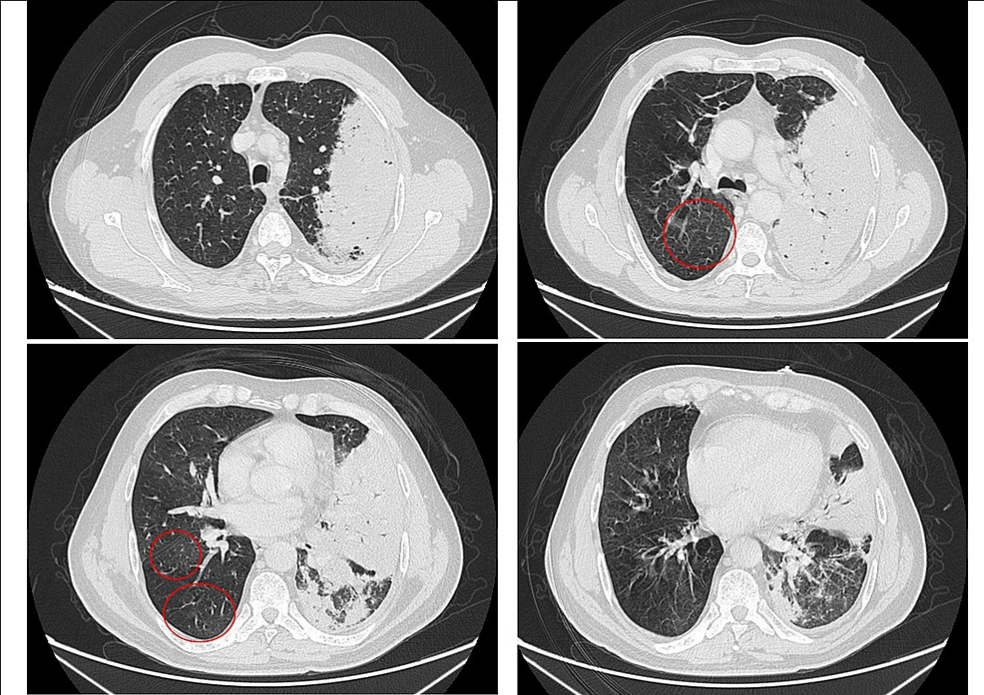
CT scan of the chest showing the opacity extending over the left lung with ground glass foci in the right lung during hospital admission

Moreover, acute respiratory distress appeared two days later, for which the patient was admitted to the emergency room. At admission, he was conscious, hypotensive, tachycardic, and polypneic. Arterial gas analysis performed while the patient was breathing in room air showed an uncompensated respiratory alkalosis with a pH of 7.428, hypoxemia (PaO2 at 53 mmHg), hypocapnia (PaCO2 at 34.4 mmHg), and saturation at 84%. He was restless, cyanotic, and sweating, with intercostal indrawing and jerky words. Bilateral sibilant rales and crackles on the left lung were found on clinical examination with no right ventricular failure signs. The rest of the clinical examinations were normal. Foci of the parenchymal condensation of the partially ventilated alveolar type at the expense of the upper lobe and the left Fowler segment with ground glass foci and a small left pleural effusion slide were found on CT scan with the injection of contrast medium without any signs of pulmonary embolism (Figure 2). Moreover, some diffuse ground glass areas in the right lung were found (the red circles in Figure 2).

The laboratory results showed hyperleukocytosis (white blood cells: 19,300/mm^3^), of which 20% were eosinophils (3,960/mm^3^), and a CRP of 375.52 mg/L. COVID-19 PCR was negative. The acid-fast bacilli smear and cytobacteriological sputum examination were negative, as was the rest of the infectious disease workup, including the cytobacteriological examination of urine (CBEU), nasal swab, and respiratory panel multiplex. Cardiac evaluation with two-dimensional echocardiogram was normal.

**Figure 3:**
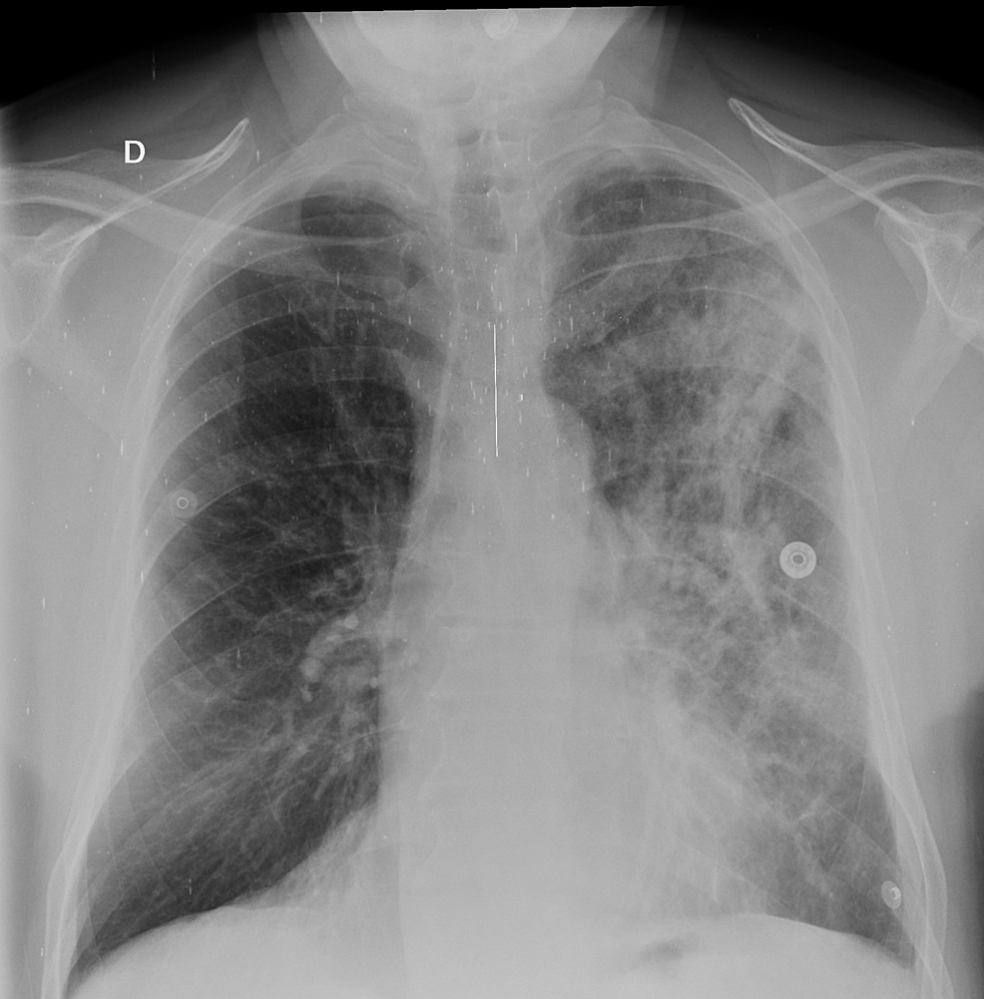
Chest X-ray showing the regression of the alveolo-interstitial syndrome in the left lung one week after the treatment

The diagnosis of acute eosinophilic pneumonia was retained. No stigmata in favor of allergic bronchopulmonary aspergillosis or vasculitis were found. Antinuclear antibodies, antineutrophil cytoplasmic antibodies, proteinase 3 antibodies, and myeloperoxidase antibodies were normal, as were total and anti-*Aspergillus* IgE. Moreover, chemical pneumonitis was excluded. Given the hypereosinophilia, the lack of clinical and biological improvement after 48 hours of antibiotic therapy, wheezing in the chest, elimination of all probable causes of hypereosinophilia, and chest X-ray, a high dose of corticosteroids was prescribed: methylprednisolone (80 mg/eight hours), combined with oxygen therapy and nebulization with salbutamol and ipratropium. An antibiotic combination was also prescribed to prevent surinfections due to the high doses of corticosteroids (a third-generation cephalosporin and quinolone). After 24 hours, the patient remarkably improved clinically. After 72 hours, white blood cells have halved. The eosinophilia disappeared, and CRP became negative. Five days after the treatment, a regression of the alveolo-interstitial syndrome was noticed on the chest X-ray (Figure 3).

A pulmonary function assessment was performed. A predominantly obstructive ventilatory disorder was found with a pretest 39% FEV-1, reversible after taking salbutamol (28% gain), with a TLC of 8.70 L in the pretest (36% gain after the reversibility test). The diffusing capacity for carbon monoxide (DLCO) was within normal limits (at 85%).

Postvaccination eosinophilic pneumonia was indeed retained due to improvement under corticosteroids and especially given the imputability of the cause-and-effect relationship with the vaccine: the patient's condition deteriorates immediately after vaccination with AZD1222.

## Discussion

Currently, no curative treatment has yet been discovered for this emerging virus. Vaccinating more than 70% of the world's population and achieving collective immunity remains the only hope. However, some risks should be considered. Immediate complications such as thromboembolic complications, including pulmonary embolism [1] and immune-induced thrombotic thrombocytopenia [2]; neurological complications, including the induction of Guillain-Barré syndrome [3]; or even allergic reactions [4], including swelling of the throat or a sensation of a foreign body in the throat [5], could occur. After a review of the literature, acute eosinophilic pneumonia has never been described in association with anti-COVID-19 vaccine, and this is the third reported case postvaccination, all vaccines combined. The first was following vaccination with the influenza vaccine (Vaxigrip*) [6] and the other after the second dose of the 23-valent pneumococcal polysaccharide vaccine (Pneumovax 23*) [7]. In all three cases, the patients were admitted in acute respiratory distress (Table 1).

**Table 1:** Comparative table of the three cases

			Case reported by Pornsuriyasak P, Suwatanapongched T, Klaewsongkram J, Buranapraditkun S, and Rotjanapan P	Case reported by Kikuchi R, Iwai Y, Watanabe Y, Nakamura H, and Aoshiba K	Case reported by Miqdadi A and Herrag M
		Vaccine	Influenza vaccine (Vaxigrip*)	Pneumococcal vaccine (polyvalent pneumococcal vaccine, Pneumovax 23*)	Anti-COVID-19 vaccine (AZD1222*)
		Year	2014	2019	2021
Age			86	68	66
History			Hypertensive arrhythmia and chronic obstructive pulmonary disease	Dialyzed following chronic glomerulonephritis	Allergic rhinoconjunctivitis
Appearance of signs			Seven days	Two days	Five hours
Clinical	General examination		Temperature, 38.7°C; respiratory rate, 26 breaths/minute; heart rate, 90 beats/minute; blood pressure, 130/80 mmHg; oxygen saturation on room air, 95%; and pO2, 48 mmHg	Temperature, 38°C; respiratory rate, 24 breaths/minute; heart rate, 102 beats/minute; and oxygen saturation on room air, 88%	Temperature, 39°C; respiratory rate, 27 breaths/minute; heart rate, 115 beats/minute; blood pressure, 104/58 mmHg; oxygen saturation on room air, 84%; and pO2, 53 mmHg
	Symptoms		Breathlessness, fever, malaise, myalgia, and a cough with scant sputum	Fever and dyspnea	Chest tightness with wheezing, polypnea, 39°C fever, asthenia, and muscle weakness
	Clinical examination		Coarse crackles and wheezes in the bilateral lower lungs	Coarse crackles in the lung regions bilaterally	Restless, cyanotic, and sweating, with intercostal indrawing and jerky words, with bilateral sibilant rales and crackles
Biological	Leukocytes in blood		15.52 x 10^9^/L	8,800/μL	19,300/mm^3^
	Eosinophils in blood		1%	11%	20%
	Leukocytes in BAL fluid		445 x 10^6^/L	9.05 x 10^5^/mL	-
	Eosinophils in BAL fluid		15%	51%	-
Radiological			Multiple patchy and/or peribronchial opacities in both lungs, notably in the right upper lobe	Diffuse infiltrative and ground glass opacities in both lung fields	Foci of parenchymal condensation of the partially ventilated alveolar type at the expense of the upper lobe and the left Fowler with ground glass foci and a small left pleural effusion slide, and diffuse ground glass areas in the right lung
Treatment			Ceftriaxone + azithromycin, 15 mg dexamethasone/eight hours, and then 60 mg prednisolone/day, and noninvasive ventilation and then mechanical ventilation	Bolus of methylprednisolone for three days and then 40 mg/day with a decrease over eight weeks	Third-generation cephalosporin and quinolone, oxygen therapy and nebulization with salbutamol and ipratropium, methylprednisolone 80 mg/eight hours, and then 40 mg/day with a decrease over 12 weeks

There was a considerable rise in eosinophils, especially in BAL fluid in the two cases described above and in blood in the case reported (knowing that the eosinophil's count in the prior was about 330/mm^3^). This can be explained by the virus itself [8,9] or the presence of allergic excipients [4], including polysorbate 80 in our case [10]. Unfortunately, we could not confirm eosinophilia in BAL fluid as the patient refused bronchial endoscopy.

Corticosteroid therapy remains the main treatment, and once introduced, clinical and later biological improvement has been noticed. The patient received corticosteroids for one month; then, a decrease over two months was performed, thus totaling three months of treatment.

## Conclusions

This case highlights the presence of a link between eosinophilic lung disease and vaccination generally and AZD1222 more specifically. Similar to any vaccine, immune reactions are possible, so it is important to notify these reactions to the local Vaccine Adverse Event Reporting System (VAERS) and monitor more closely those who may develop similar reactions. As the exact link has not been identified with certainty and in cases of severe allergies or in patients with allergy or asthma who are poorly monitored, the use of another less allergenic vaccine could be considered. Moreover, a monitoring system should be proposed to these pulmonary fragile patients after vaccination to handle quickly any potential side effects. However, this should in no way prevent the continuation of the vaccination given the benefit incurred.
